# *B. anthracis* associated cardiovascular dysfunction and shock: the potential contribution of both non-toxin and toxin components

**DOI:** 10.1186/1741-7015-11-217

**Published:** 2013-10-09

**Authors:** Kenneth E Remy, Ping Qiu, Yan Li, Xizhong Cui, Peter Q Eichacker

**Affiliations:** 1 Critical Care Medicine Department, Clinical Center, National Institutes of Health, Bethesda, MD 20892, USA

**Keywords:** *Bacillus anthracis*, Anthrax, Cell wall components, Lethal and edema toxins, Metalloproteases, Cardiovascular dysfunction, Shock

## Abstract

The development of cardiovascular dysfunction and shock in patients with invasive *Bacillus anthracis* infection has a particularly poor prognosis. Growing evidence indicates that several bacterial components likely play important pathogenic roles in this injury. As with other pathogenic Gram-positive bacteria, the *B. anthracis* cell wall and its peptidoglycan constituent produce a robust inflammatory response with its attendant tissue injury, disseminated intravascular coagulation and shock. However, *B. anthracis* also produces lethal and edema toxins that both contribute to shock. Growing evidence suggests that lethal toxin, a metalloprotease, can interfere with endothelial barrier function as well as produce myocardial dysfunction. Edema toxin has potent adenyl cyclase activity and may alter endothelial function, as well as produce direct arterial and venous relaxation. Furthermore, both toxins can weaken host defense and promote infection. Finally, *B. anthracis* produces non-toxin metalloproteases which new studies show can contribute to tissue injury, coagulopathy and shock. In the future, an understanding of the individual pathogenic effects of these different components and their interactions will be important for improving the management of *B. anthracis* infection and shock.

## Introduction

Recent outbreaks of *Bacillus anthracis* infection in the United States (US) and Europe have underscored the importance of this bacterium in the developed world [1-4]. Despite aggressive support, mortality rates in these outbreaks have been high; 40% in the 2001 US outbreak of inhalational infection, and 33% in the 2009 outbreak of injectional disease in Scotland [2,4,5]. During these outbreaks, the development of shock in patients has appeared resistant to standard hemodynamic therapy and has been associated with a particularly poor prognosis [1-3]. Therefore, an increased understanding of the mechanisms producing shock during *B. anthracis* infection will be important for its management.

Evolving research has shown that *B. anthracis* produces several components potentially important in the pathogenesis of shock, including its two exotoxins (lethal and edema toxins (LT and ET, respectively)), a cell wall and its constituents, and several non-toxin metalloproteases. Notably, although LT and ET have long been a focus in this area of research, newer data have begun to emphasize the role of these non-toxin components. This review highlights recent research directed at the contribution of these different non-toxin and toxin components.

### *B. anthracis* cell wall and its peptidoglycan constituent

Sepsis is thought to typically start as a nidus of infection, followed by bacterial invasion of the blood stream. Bacterial cell wall and other components interact with pathogen recognition receptors (PRRs) on host cells; host defense systems are activated; and inflammatory mediators (for example, cytokines, nitric oxide and oxygen free radicals) are released [6]. While this inflammatory response is an essential part of innate immunity and is necessary for microbial clearance, an excessive response can produce organ injury and shock [7-9]. Growing evidence suggests that *B. anthracis* infection can elicit this type of maladaptive injurious host inflammatory response (Figure?1A) [10,11].

**Figure 1:**
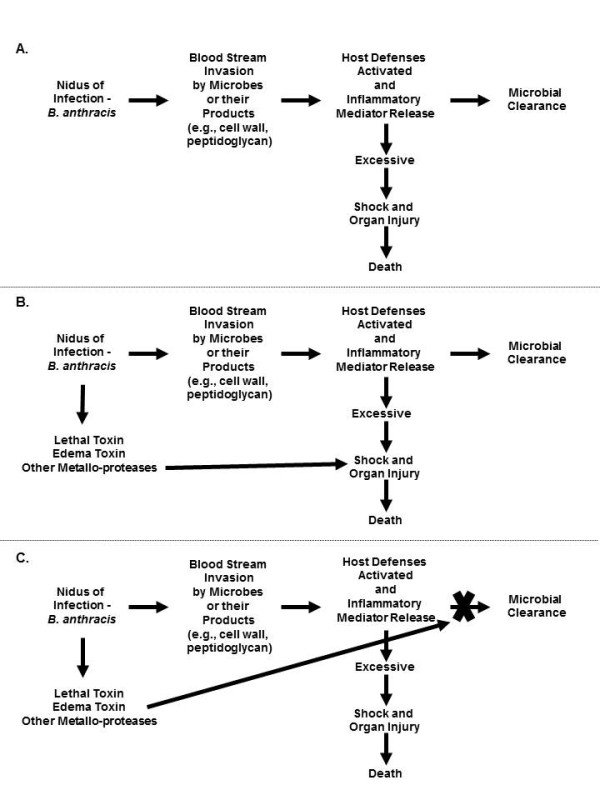
**Overview of basic pathways potentially leading to shock, organ injury and death during *B. anthracis *infection. A**. As Gram-positive bacteria, *B. anthracis* and its products (for example, cell wall and peptidoglycan) activate host defenses and inflammatory mediator release which are necessary for microbial clearance. However, if this response is excessive it may result in the development of shock, organ failure and death. **B**. *B. anthracis* also produces two exotoxins, lethal and edema toxins, which are capable of contributing directly to shock, organ injury and death via diverse mechanisms. **C**. Lethal and edema toxin also appear capable of subverting critical host defense systems and contributing to the pathogenesis of shock, organ injury and death by limiting microbial clearance. Other mechanisms not depicted in this figure, such as the activation of metalloproteases other than lethal factor, may contribute to shock and organ injury with *B. anthracis* as well (see text).

While lethal and edema toxins are now recognized not to stimulate excessive inflammation (and may actually suppress it), the vegetative form of *B. anthracis* has a cell wall comprised largely of peptidoglycan that can promote such inflammation [12-14]. Whole cell wall and/or purified peptidoglycan can interact with toll-like receptors two and six (TLR2/6) and nucleotide-binding oligomerization domains 1 and 2 (NOD1 and 2) proteins [15,16]. They can also stimulate host release of TNF?, IL-1? IL-6 and other inflammatory mediators [17]. In rats, in contrast to LT or ET, purified *B. anthracis* cell wall or its peptidoglycan component alone can stimulate a robust inflammatory response with resultant tissue injury, shock, disseminated intravascular coagulation and lethality [18,19]. Relevant to these findings, *B. anthracis* peptidoglycan can activate platelets through complement activation [20]. The potential pathogenic role of cell wall takes on added importance based on the observation that patients and animals dying from *B. anthracis* frequently have very high bacterial loads [2,13,19]. Therefore, while much attention has been devoted to the unique roles LT and ET have in the pathogenesis of *B. anthracis*, this Gram-positive bacteria can also produce shock and organ injury via mechanisms common to other types of bacteria.

### Lethal and edema toxin structure and intracellular effects

Lethal and edema toxins are each binary type exotoxins comprised of protective antigen (PA) and the toxic moieties lethal factor (LF) and edema factor (EF) [14,21,22]. During infection, PA binds to host cells via one of two receptors: tumor endothelial marker 8 (TEM8) or capillary morphogenesis gene-2 (CMG2) [23-25]. These receptors are abundant on endothelial cells and in a variety of tissues [14]. PA binding is necessary for host cell uptake of LF and EF. LF inactivates mitogen-activated protein kinase kinases 1 to 4, 6 and 7 (MAPKKs) [21,22]. Recent data also show that LF activates the Nlrp1 inflammasome in macrophages and dendritic cells causing activation of caspase-1 and the production of IL-1? and IL-18 and, subsequently cell death [26-28]. EF has potent calmodulin-dependent adenyl cyclase activity and increases intracellular concentrations of cyclic adenosine monophosphate (cAMP) and possibly other cyclic nucleotides [29,30]. While neither toxin stimulates a robust inflammatory response, growing evidence demonstrates that each can contribute to cardiovascular dysfunction and shock (Figure?1B).

### Lethal toxin

Studies in an instrumented canine model clearly demonstrated that LT produces profound cardiovascular dysfunction. Twenty-four-hour LT infusions to simulate toxin release during infection resulted in reductions in central venous pressure (CVP) and mean arterial blood pressure (MAP) that persisted for up to 72 hours [31,32]. These changes occurred in patterns similar to those in patients with *B. anthracis* infection and shock; and were greater in non-survivors than survivors. Hemodynamic effects such as these frequently reflect peripheral vascular dysfunction related either to endothelial barrier dysfunction with extravasation of fluid or direct dilation of arterial resistance or venous capacitance vessels. Consistent with such effects, administration of fluids and vasopressors in this LT-challenged model increased CVP, MAP and survival [32]. Inhibition of LT with a PA-directed monoclonal antibody (mAb) further increased these parameters. A growing number of *in vitro* studies provide a basis for these changes with LT. Table?1 briefly summarizes findings from recent studies demonstrating that LT may interfere with endothelial barrier function via several mechanisms including: disruption of endothelial cell stress kinase pathways, endothelial apoptosis, and alterations in actin fiber and cadherin function [33-36]. However, in contrast to its potential deleterious effects on endothelial barrier function, LT did not produce direct arterial dilation in an isolated aortic ring model [37].

**Table 1:** **Selected *in vitro *** **studies implicating lethal (LT) or edema toxin (ET) in endothelial cell dysfunction**

**Study**	**Toxin**	**Cell type**	**Toxin effect**
Rolando, M. 2010	LT	HUVEC	Exerted cytotoxic effects on endothelial cell monolayers with elongation and redistribution of VE-cadherin and subsequent cell death; increased caspase-3, 8 and 9 activity. Up-regulation of TNF-related apoptosis-inducing ligand (TRAIL) and down-regulation of xaf1 (XIAP associated factor-1) participated in LT-induced caspase-3 activation; increased caspase-3 dependent cortactin and rhophilin-2 activity in combination with calponin-1 expression appeared necessary for LT mediated actin cable formation.
Guichard, A. 2010	LT	Human brain, dermal and lung microvascular endothelial cells (HBMEC, HDMEC and HMVEC-Ls, respectively)	Lethal factor (LF) worked synergistically with edema factor (EF) to reduce DE-cadherin levels at adherens junctions in HBMEC, HDMECs and HMVEC-Ls.
Warfel, J. 2011	LT	Human lung microvascular endothelial cells	Increased monolayer permeability, effects on permeability associated with the activation of Rho associated kinase (ROCK-1) and increased myosin light chain (MLC) phosphorylation and subsequent actin stress fiber formation and VE-cadherin gene and protein expression inhibition.
Liu, T. 2012	LT	Rat pulmonary microvascular endothelial cells	Increased gap formation and permeability of endothelial cell monolayers; decreased p38 signaling; permeability effects overcome by pmHSP27 over-expression.
Guichard, A. 2010	ET	Human brain, dermal and lung microvascular endothelial cells (HBMEC, HDMEC and HMVEC-Ls, respectively)	Edema factor (EF) worked synergistically with lethal factor (LF) to reduce DE-cadherin levels at adherens junctions in HBMEC, HDMECs and HMVEC-Ls.
			EF increased the permeability of HBMEC trans-well monolayers.
Maddugoda, M. 2011	ET	Mouse endothelial cells, HUVEC	Stimulated trans-endothelial macro-aperture (TEM) tunnel formation and increased endothelial permeability potentially via cAMP mediated mechanisms.
Ebrahimi, C. 2011	ET	HBMEC	Disrupted tight junction formation and barrier function and monolayer integrity; contributed to disruption of endothelial cells and ZO-1, a primary regulatory protein of tight junction formation in the blood?brain barrier.

Evidence also suggests that LT depresses myocardial function. In canines, a 24-hour LT infusion caused progressive decreases in left ventricular ejection fraction (LVEF) that were blocked by PA-mAb [31,32]. Although CVP was reduced, pulmonary artery occlusion pressure was not, suggesting that decreased preload was not the primary basis for LVEF reductions. Similar to mice, more recent work in rabbits demonstrated that LT produced direct myocardial injury with dose dependent cardiac necrosis and increases in cardiac biomarkers [38,39]. In *in vitro* studies, LT altered intracellular cardiomyocyte-Ca^++^ handling and depressed cardiomyocyte function [40]. LT associated dysregulation of autophagy, ubiquitin-proteasome, and mitochondrial function may have contributed to this cardiomyocyte depression via TLR-4 [41,42]. Thus, hypotension in patients with *B. anthracis* may also relate to the inhibitory effects of LT on cardiac function. However, in contrast to data supporting a myocardial depressant effect of LT in studies in an isolated perfused rat heart model, LT only altered myocardial function when administered in doses substantially higher than those producing lethality *in vivo*[43]. Furthermore, observations in patients with *B. anthracis* infection, while limited, have not demonstrated consistent abnormalities in myocardial function [1-3].

In addition to its direct cardiovascular effects, LT may contribute to shock by promoting *B. anthracis* infection [44]. LT inactivates MAPKK pathways central to innate and adaptive immune responses and, therefore, may impair host defense and microbial clearance [22,25,45-47]. In one murine model, pretreatment with sublethal LT doses before intravenous *E. coli* challenge, increased blood bacterial counts and worsened survival [48]. Moreover, pretreatment of rats with sub-lethal LT doses inhibited inflammatory mediator release stimulated by lipopolysaccharide (LPS) or *E. coli* challenges [14]. These inhibitory effects of LT on immune responses have been proposed as a basis for the high bacterial loads noted in patients dying with *B. anthracis* infection [13,19,21,22].

### Edema toxin

Increasing evidence indicates that ET may also be important in the pathogenesis of shock during *B. anthracis* infection. Twenty-four-hour ET challenge in canines produced rapid and profound reductions in CVP, MAP and systemic vascular resistance (SVR) that persisted for 72 hours [31]. Recent *in vitro* studies, summarized in Table?1, suggest that ET may impair endothelial barrier function by altering adherens? junction function or by inducing trans-endothelial macro-aperture tunnels [36,49,50]. However, endothelial impairment is not entirely consistent with EFs? recognized action as a potent adenyl-cyclase, since increased cellular cAMP levels may actually have protective effects on endothelial barrier function [12,51,52]. Consistent with this, ET in one study increased endothelial barrier resistance [53]. Alternatively, very high cAMP levels and their intracellular location may have paradoxical effects on endothelial integrity resulting in a net loss of barrier function [12,51,52].

Growing data suggests that EFs? potent adenyl cyclase actions may stimulate direct arterial or venous dilation. It is well known that increased intracellular cAMP levels stimulate vascular smooth muscle relaxation [54,55]. The very rapid reductions in CVP, MAP and SVR noted with ET in the canine model were consistent with a direct vasorelaxant effect rather than with disruption of endothelial barrier function and extravasation of fluid [31]. Findings from two *ex vivo* models further support this possibility. In an isolated perfused rat heart model, ET produced significant increases in coronary flow rate (CFR) consistent with a direct vasodilatory effect [43]. These changes with ET were associated with increases in both myocardial tissue and effluent cAMP levels. Adefovir, a nucleoside which interferes with EF adenyl-cyclase activity, inhibited these ET effects [56,57]. In a rat aortic ring model, incubation with ET increased cAMP levels and reduced arterial responsiveness to subsequent contraction with phenylephrine [37]. ET also caused relaxation in rings already pre-contracted with phenylephrine. This ability of ET to inhibit catecholamine function may provide a basis for the resistance to conventional hemodynamic support noted in patients with injectional *B. anthracis* infection [1-3].

Finally, ET may augment the effects of LT. In mouse, rat and canine models, nonlethal ET doses increased the lethality of LT [14,31]. ET also potentiated the inhibitory effects of LT on chemotaxis and the function of dendritic and T-cells [14]. ET?s ability to up-regulate the expression of PA receptors on macrophages and dendritic cells *in vitro* and to increase the rate of toxin internalization may provide a basis for synergism with LT [58]. Growing recognition of ET?s potential role in the pathogenesis of shock with *B. anthracis* suggests that if toxin-directed therapies are to be considered for patients, they should be directed at both LT and ET [2].

### Non-toxin metalloproteases

Besides LF, *B. anthracis* produces other metalloproteases potentially important in the pathogenesis of vascular and nonvascular tissue injury. The delta Ames (pXO1^-^ and pXO2^-^) *B. anthracis* strain produces metalloproteases belonging to the M4 thermolysin and M9 bacterial collagenase families [59,60]. In murine models, administration of these metalloproteases produced hemorrhagic tissue injury while treatment with selective metalloprotease-inhibitors improved survival [61,62]. *B. anthracis* metalloproteases Npr599 and InhA cleaved host structural and regulatory proteins important in endothelial function, including plasma ADAMTS13, von Willebrand factor (VWF) substrate FRETS-VWF73, and VWF itself [63]. InhA also stimulated plasminogen activator inhibitor (PAI-1) in mouse liver and increased blood?brain barrier permeability in both mouse brain and human brain microvasculature endothelial cells by disrupting endothelial tight junction proteins [61,62,64,65]. What the relative roles are of LF and these other metalloproteases during shock in patients with *B. anthracis* infections require study.

## Conclusions

There is growing evidence that the pathogenesis of cardiovascular dysfunction and shock during *B. anthracis* infection is complex and likely involves both non-toxin and toxin components. Further understanding of how these components interact is essential for improving the management of severe *B. anthracis* infection and shock.

## Abbreviations

cAMP: Cyclic adenosine monophosphate; CFR: Coronary flow rate; CMG-2: Capillary morphogenesis gene-2; CVP: Central venous pressure; EF: Edema factor; ET: Edema toxin; HBMEC: Human brain microvascular endothelial cells; HDMEC: Human dermal microvascular endothelial cells; HMVEC-L: Human lung microvascular endothelial cells; HUVEC: Human umbilical vein endothelial cells; IL: Interleukin; LF: Lethal factor; LPS: Lipopolysaccharide; LT: Lethal toxin; LVEF: Left ventricular ejection fraction; MAPKinase: Mitogen activated protein kinase; MLC: Myosin light chain; NLRP: Nod Like Receptor Protein; NOD: Nucleotide-binding oligomerization domains; PA: Protective antigen; PAI-1: Plasminogen activator inhibitor; PRRs: Pathogen recognition receptors; ROCK-1: Rho associated kinase; SVR: Systemic vascular resistance; TEM: Trans-endothelial cell macro-aperture; TEM-8: Tumor endothelial marker-8; TLR: Toll like receptor; TNF: Tumor necrosis factor; TRAIL: TNF-related apoptosis-inducing ligand.

## Competing interests

None of the authors have competing interests to report.

## Authors? contributions

All authors (KER, PQ, YL, XC and PQE) have contributed to the formulation, writing and editing of this review. All authors read and approved the final manuscript.
